# Development of Tailored Porous Ti6Al4V Materials by Extrusion 3D Printing

**DOI:** 10.3390/ma18020389

**Published:** 2025-01-16

**Authors:** Luis Olmos, Ana Silvia González-Pedraza, Héctor Javier Vergara-Hernández, Didier Bouvard, Monserrat Sofía López-Cornejo, Rumualdo Servín-Castañeda

**Affiliations:** 1 Universidad Michoacana de San Nicolás de Hidalgo, INICIT, Fco. J. Mujica S/N, Morelia 58060, Michoacán, Mexico; luis.olmos@umich.mx; 2 División de Estudios de Posgrado e Investigación, Tecnológico Nacional de México/I.T. Morelia, Av. Tecnológico #1500, Colonia Lomas de Santiaguito, Morelia 58120, Michoacán, Mexico; hector.vh@morelia.tecnm.mx (H.J.V.-H.); monserrat.lc@morelia.tecnm.mx (M.S.L.-C.); 3 University Grenoble Alpes, CNRS, SIMAP, 38000 Grenoble, France; didier.bouvard@grenoble-inp.fr; 4 Universidad Autónoma de Coahuila, Facultad de Ingeniería Mecánica y Eléctrica Unidad Norte, Monclova 25720, Coahuila, Mexico; rumualdo.servin@uadec.edu.mx

**Keywords:** additive manufacturing, porosity, computed tomography, permeability, sintering

## Abstract

Nowadays, metallic bone replacement is in high demand due to different issues, like sicknesses and accidents. Thus, bone implants are fabricated with tailored properties and microstructure for long-term use in the human body. To improve such implants, 3D printing is the most promising technique. Therefore, this work aims to evaluate the fabrication of porous materials by extrusion 3D printing of Ti6Al4V. Cylindrical samples were fabricated from pellets for metal injection molding of Ti6Al4V powders, creating hexagonal channels with three different sizes. The densification kinetics was evaluated by dilatometry tests, which enabled following the densification of the samples during the sintering cycle. Subsequently, the samples were characterized by scanning electron microscopy and X-ray computed tomography to analyze their microstructure. Compression tests evaluated the mechanical strength of sintered samples. It was found that the hexagonal shape during printing is better defined as the channel size increases. The results show similar behavior for each of the channel sizes during sintering; however, greater densification is obtained as the channel size decreases. Additionally, microporosity is obtained at the particle level, which is completely interconnected, ensuring the passage of fluids through the entire sample. On the other hand, as the channel size increases, Young’s modulus and yield strength are considerably reduced. The main conclusion is that parts with two scales of porosity can be designed by the 3D printing extrusion process.

## 1. Introduction

In biomedical implants, porosity is a property that should not go unnoticed because it is through porosity that the diffusion of nutrients and vascularization occurs [1,2]. Besides improving the passage of fluids, porosity helps decrease mechanical properties to avoid the so-called “stress shielding.” This is due to the high difference between the modulus of commonly used materials such as Ti6Al4V (Ti64) (90–115 GPa) [3], Co alloys (210–250 GPa) [4], and stainless steel (200 GPa) [5] compared to human bone (cortical bone 7–30 GPa [6]).

Different techniques have been developed for the fabrication of porous materials in order to reduce mechanical properties [7] and, at the same time, achieve better tissue–implant bonding [8]. To improve biomedical applications, porous materials classified as open cell are preferred because they show excellent interconnectivity and low mechanical resistance [9]. Among the techniques used to fabricate porous materials, the space holder technique is one of the most commonly used, in which particles with lower melting points are used as spacers. The pores’ features depend on the shape, size, and quantity of space holders added. This technique’s main advantage is the possibility of fabricating porous composites that can improve biocompatibility [10,11,12]. Karakurt et al. [13] fabricated a porous alloy (21–25% and 50–56%) of Ti-Nb-Zr to study the corrosion effect and in vitro tests. During the corrosion tests, they used Hank’s solution, demonstrating better corrosion resistance for the alloys with low porosity (21–25%) in the same way they presented higher cell growth and adhesion. At the same time, the mechanical tests showed a low Young’s modulus as the porosity percentage increased (50–56%). Another technique to consider for forming open-cell pores is through a liquid-state process, i.e., injecting gas (argon, air, nitrogen) directly into the molten metal and thus creating bubbles inside the metal and obtaining the porosity after cooling. Using this technique, the porosity can be controlled through the gas flow and the nozzle diameter, among others. However, controlling the pore size and morphology is impossible since only sub-spherical pores are obtained [14].

On the other hand, additive manufacturing (AM) has presented advantages over porous scaffold fabrication because it is possible to control the porosity while designing pores with complex architectures. Li et al. [15] fabricated Ti64 scaffolds by melting with pore sizes between 300 and 500 µm in four different morphologies. They demonstrated that as the channel size increases, the modulus is considerably reduced, as well as the role that channel morphology can play. On the other hand, they found that cell size and morphology play an important role in cell growth due to more cells for a size of 300 µm, with osseointegration and permeability being important factors in the fabrication of porous materials. Ouyang et al. [16] demonstrated how pore size influences the results of these properties as they fabricated titanium porous scaffolds by selective laser melting (SLM) with four different pore sizes (400, 650, 850, 1000 µm), finding that as pore size increased it favored permeability, allowing fluid entry and flow velocity. However, in vitro, results showed that a large pore size allowed cell penetration while smaller pore sizes favored cell deposition. Finally, during in vivo tests, they found that a medium pore size (650 µm) better supports bone regeneration than large pore sizes. Chen et al. [17] corroborated that pore size influences cell proliferation, osteogenesis, and bone growth. They found that smaller pore size and volume pore fraction results in better cell adhesion, proliferation, and osteogenic differentiation. Vega et al. [18] developed Ti coatings that were applied to cylindrical Ti64 endomedullary systems for evaluation in in vivo tests. They found that roughness surfaces significantly improved the cancellous bone growth around the implant, as well as good tissue–implant fixation. Deering et al. [19] fabricated porous scaffolds of 600 and 300 µm of Ti64 through SLM and performed osseointegration tests in tibiae rabbits. Through tomography, they observed incredible bone growth not only on the outside but also on the inside of the scaffolds; they found that the optimal pore size for osseointegration is 300 µm because it presented a more significant growth in 12 weeks. Another of the most widely used techniques is selective laser sintering (SLS) since it is possible to fabricate metallic scaffolds that aid bone regeneration [20]. Because of this versatility, SLS has been used to fabricate scaffolds with mechanical properties close to those of trabecular bone [21]. On the other hand, one of the main disadvantages is that the high sintering temperatures limit the inclusion of cells and biomaterials in the scaffolds [22], as well as restrict the pore size due to the characteristics of the powders, such as particle size.

Although laser techniques are outstanding, the main disadvantage is that pores are more significant than hundreds of microns, which, as pointed out above, reduces the mechanical properties but diminishes the osseointegration abilities of implants. To overcome such disadvantages, extrusion 3D printing offers an alternative to control the porosity in the parts besides larger designed pores [23,24]. This is thanks to the process involving a sintering step, which can control interparticle porosity by the thermal cycle. Among the different extrusion 3D printing technologies, stereolithography (SLA) was one of the first printing techniques for bone engineering. This is due to its precision in producing structures with nanometer and micrometer scales, in addition to having the ability to fabricate complex shapes with internal architectures and to obtain extremely high resolution (1.2 µm). However, the number of materials used in bone engineering is limited due to viscosity, stability, and refractive index restrictions.

In some cases, cytotoxic effects may be caused by irradiation with ultraviolet light [25]. Another extrusion technique is the Fused Deposition Modeling (FDM-extrusion), in which there is a sub-classification of the same depending on the type of feed that has the equipment (pellets, filaments, or bars), but they have similar procedures. This technique has more advantages over the other technologies since it is possible to control the printing parameters, such as temperatures, nozzle anchor and angle, and printing directions, which help to manufacture and control scaffolds with the desired pore size, morphology, and interconnectivity [26]. This is why the extrusion technique can produce complex 3D parts that are difficult to fabricate for other technologies (lithography and micromachining). Studies have shown that scaffolds fabricated by FDM provide favorable biochemical and mechanical properties for bone regeneration [27,28,29,30].

Nowadays, AM has emerged to develop the regenerative medicine field that aims to repair or replace damaged tissues and organs. Concerning bone implants, bone tissue engineering seeks to restore or regenerate damaged or lost bone tissue. This approach is focused on combining materials engineering with biology to generate bone implants that can better mimic bone structure and function [31,32]. As such, “active” scaffolds have been developed to serve as structures over the which the natural bone tissues will be grown and, in some cases, this structure is absorbed by the natural bone, accelerating patient recovery [33]. To improve the scaffolds, most studies are focused on controlling the porosity, which plays the major role in the new bone growth [34]. Thanks to AM, it was possible to evaluate the effect of the pore size as well as interconnectivity to the regenerative process [35,36]. In such works, it was demonstrated that pore size has a strong influence on cell adherence and interconnectivity in cell proliferation [37]. In the same way, it was shown that large pores favor vascularity and promote high permeability, which can help pass through corporal fluids with nutrients and dispose waste from the organism [38]. Further, the porosity controls the mechanical properties discussed above, thus most research is focused on developing different pore configurations. Li et al. [15] evaluated the effect of pore shape on the cell viability of titanium scaffolds, finding that a honeycomb shape induced the highest cell regeneration and the highest mechanical strength among the different shapes tested.

According to the background above, this work focuses on fabricating tailored porous materials with hexagonal channels fabricated by extrusion 3D printing of Ti64 pellets. The effect of the size of designed channels on the densification and microstructure is analyzed by SEM and X-ray microtomography. Young’s moduli are obtained through compression tests to determine the most optimal for biomedical implants. Finally, permeability is evaluated by numerical simulations using authentic 3D images.

## 2. Materials and Methods

### 2.1. Raw Material

**Figure 1:**
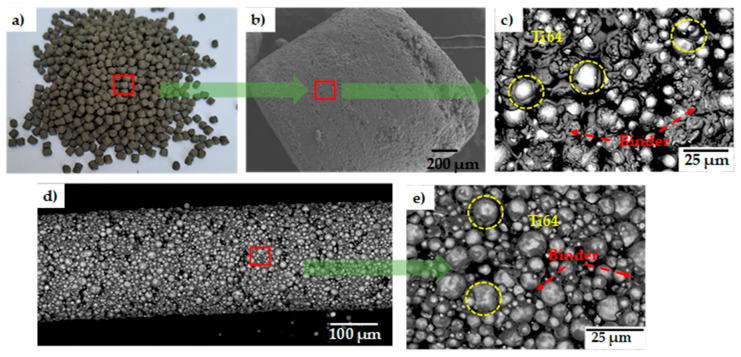
Ti64 raw material: (**a**) pellets, (**b**) pellet seen from SEM, (**c**) inside of pellet, (**d**) SEM micrograph of the filament obtained from print extrusion, and (**e**) larger magnification inside of filament.

Figure 1 shows the raw material used to fabricate the porous materials, and Figure 1a–c, illustrates the Ti64 feedstock form, which is poured into the extruder of the AIM3D printing machine (Rostock, Germany). Pellets are approximately 1 mm in diameter and 1 mm in height, furnished by PolyMIM (Bad Sobernheim, Germany). This feedstock consists of granules containing 93.5 wt.% of Ti64 powders and two polymers, namely polyethylene glycol (PEG) and some wax (not precisely specified by the provider). PEG offers flexibility during printing due to its thermoplastic nature. Figure 1c shows how the Ti64 particles are randomly embedded in the binder. From those pellets, the extrusion is performed with a nozzle of 400 µm in diameter, and the filament obtained is shown in Figure 1d. At low magnification, Ti64 particles of different sizes are observed; however, at higher magnification, the presence of the binder around the Ti64 particles is observed as shown in Figure 1e. It can be seen that the distribution of the binder is modified from its original arrangement inside the pellets.

### 2.2. Printing Parameters

**Figure 2:**
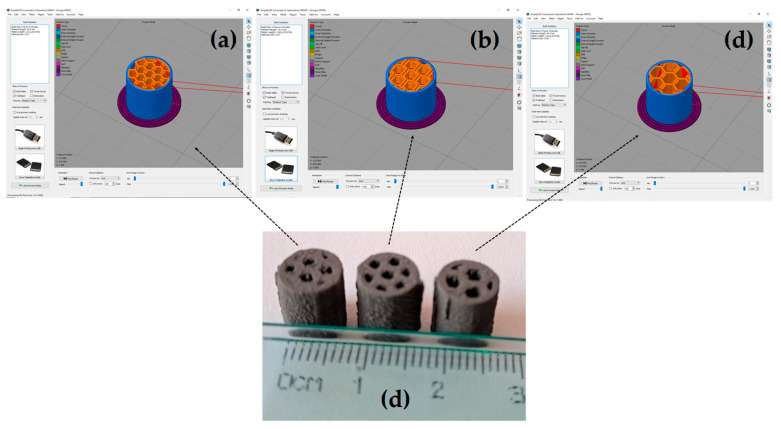
Images of the designed samples using the Simply 3D software: (**a**) small canals, (**b**) mediums canals, (**c**) large canals and (**d**) the resulting printed samples for each canal size.

Cylindrical samples of 8 mm in diameter and 10 mm in height were created in a CAD model using the SolidWorks software (2022 version). Then, the model was imported to the Simply 3D program (Version 4.0), which connects to the 3D printer. A hardened steel nozzle of 400 µm in diameter was placed at the extruder end for the filament extrusion. The impression parameters used had a layer thickness of 0.05 mm, a nozzle speed of 20 mm/s, an extrusion flow rate of 120% of the standard value, an extrusion temperature of 196 °C, and a bed temperature of 60 °C. The impression parameters were selected from previous works devoted to optimizing the full density of parts by Singh et al. [39]. They used MIM pellets of copper furnished by the same company and the same 3D printer. Additional details on the printing parameters can be found elsewhere [39,40,41]. To create porous samples, the Simply 3D software offers the possibility to develop trajectories that can make geometrical figures, like squares, triangles, and honeycomb shapes. Therefore, cylindrical samples were printed using the honeycomb model. A honeycomb shape is made by hexagons of the same size homogeneously distributed in the cylinder. In order to evaluate the size of the channels, honeycomb shapes were designed to be of three different sizes; from here, those samples will be called small, medium, and large. All samples were programmed to fill 80% of the total volume of cylinders, which means that channels are homogeneously distributed in the sample. Figure 2 shows the trajectories and samples designed in Simply 3D and the printed 3D cylinders. As can be seen, some defects are obtained because of the filament trajectory. However, the size of channels was different in each sample, as intended in this work.

### 2.3. Debinding and Sintering

After printing green parts, they must be subjected to a binder elimination process, which consists of two stages, one in solvent and the other thermal, as indicated in the literature [42]. For solvent elimination, the parts were subjected to distilled water at a temperature of 60 °C for 12 h, as indicated by Singh et al. [40], so that this would form interconnected paths throughout the sample (backbone) to help in the release of the binder (in the form of gas) during thermal elimination [43]. A Linseis L75 vertical dilatometer (Selb, Germany) with a controlled argon atmosphere was used for thermal removal. A heating ramp of 10 °C/min up to 500 °C with a dwell time of 10 min was carried out, which is supported in the study of Gonzalez et al. [44] for extrusion printed parts of similar feedstock. Consequently, within the dilatometer, the sintering process was performed, where a second ramp was added, which consisted of a heating rate of 20 °C/min up to a temperature of 1100 °C with a dwell time of 1 h. This temperature was chosen in order to control the dedensification of powders to obtain interparticle porosity (void space in between the particle packing) inside the struts. As reported before, different pore sizes help to improve the adhesion and growth of osteoblast cells, which promotes bone ingrowth [1]. Therefore, it is needed to have interconnected porosity that is reached by staying in the intermediate stage of sintering, according to the sintering theory [45]. Similar thermal cycles were used for solid samples by Su et al. [46] for Ti64 parts manufactured by MIM, in which densities of 66.59% were obtained, as well as by Cabezas et al. [47], where Ti64 parts were sintered by powder metallurgy, obtaining densities of 60%. Finally, the samples were cooled at 25 °C/min to room temperature under an argon atmosphere. Thus, the sintering thermal cycle was similar to obtain a porosity of approximately 35% inside the struts because the pellets contain Ti64 powders with a similar particle size distribution, which ensures that densification will be similar under the same sintering parameters.

### 2.4. Sample Characterization

3D images of printed samples were acquired before sintering with a Zeiss Xradia 510 Versa 3D X-ray microscope (Jena, Germany) with a voxel resolution of approximately 8 µm to observe the whole sample. After sintering, 3D images were also acquired using the exact voxel resolution to observe the changes in the channels. Further, samples were increased to 1.5 mm diameter, and additional 3D images were acquired with a voxel resolution of 1.5 µm to observe the microstructure at the particle scale.

After that, the porous samples were cut in half and subjected to metallographic preparations. The surface was roughened using SiC paper in ascending order, followed by mirror polishing with alumina powders with a particle size of 1 µm. Finally, they were subjected to an ultrasonic bath for 30 min to remove any residue or impurities. The microstructure was then observed with a JEOL JSM-5910LV scanning electron microscope (Tokio, Japan).

The 3D image process was carried out using the Avizo^®^ software (V9.0) and the Image J software (https://imagej.net/ij/, accessed on 12 January 2024). The solid part channels were segmented using a thresholding procedure based on the gray level of the voxels, which is determined by the absorption coefficient of each phase [48]. This procedure is detailed by Olmos et al. [49]. The same thresholding process was performed for each channel separately (in the samples before and after sintering) in 2D to obtain the area in each channel before and after sintering. Through filters (opening and closing), the images of each channel were obtained, eliminating separate objects that were not part of the channels. Finally, the area was calculated along the sample with a label analysis where the calculation of the area in each of the slices of the image is indicated.

The numerical simulations of permeability were performed using the Avizo software in the 3D images of samples. Two different runs were performed, one in the channels of the sample and the second in the solid part, taking into account interparticle porosity. Human blood viscosity was used at 0.045 Pa s to make a more realistic simulation. The solution was obtained by solving the Navier–Stokes equations described elsewhere [50].

### 2.5. Mechanical Properties

Compression tests were conducted using an Instron 1150 universal machine, which provided a strain rate of 0.5 mm/s, as indicated in ASTM-D695-02 [51]. The elastic limit and Young’s modulus (E) were calculated from the stress–strain data obtained by the equipment. Three different samples were tested, and the average values were taken.

## 3. Results

### 3.1. Sintering Analysis

**Figure 3:**
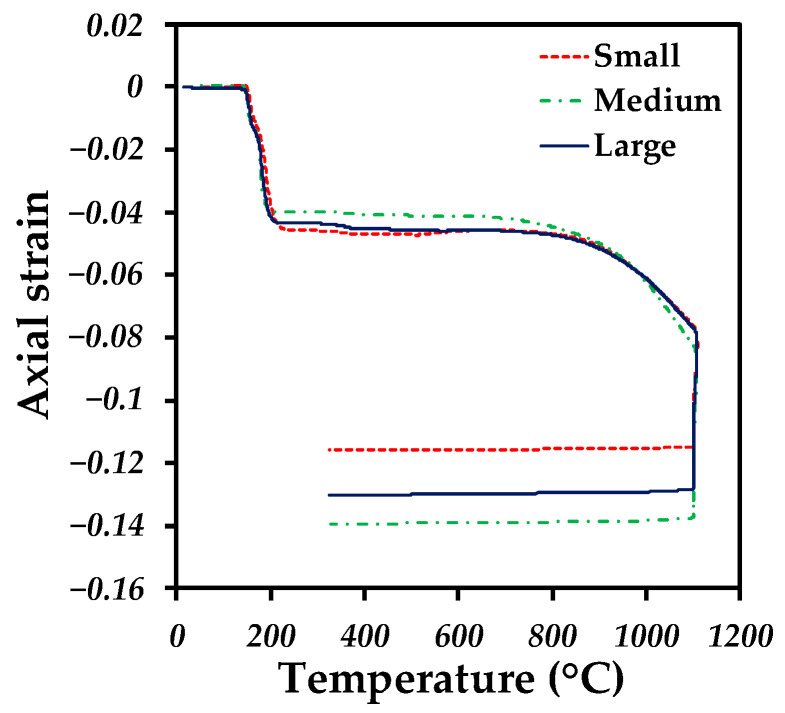
Axial strain as a function of temperature during the sintering cycle for samples printed with different sizes of honeycomb channels.

Dilatometry was used to obtain axial deformation during the sintering thermal cycle for each sample with different channel sizes. Figure 3 depicts the deformation as a function of temperature; at the beginning of the process, a constant behavior is observed for each sample. However, as the temperature increases, a negative axial deformation (shrinkage) is observed from 140 °C, which is associated with the elimination of the binder up to 240 °C, indicating the total elimination of the binder. Then, with the temperature increase, no dimensional changes are observed for any sample up to a temperature of 650 °C, which indicates the beginning of sintering. During the sintering stage, continuous shrinkage is undergone until the isothermal period is reached. A linear shrinkage is noticed during this stage because it is obtained at a constant temperature. Finally, a final shrinkage is observed due to thermal contraction during cooling. A higher deformation is obtained for the sample with a medium channel size, followed by the large and small channels.

**Figure 4:**
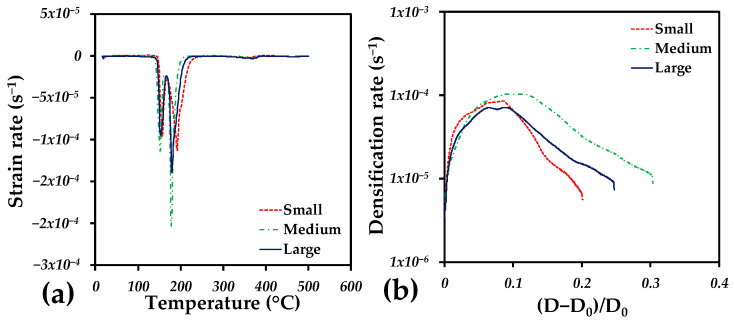
(**a**) The strain rate as a function of temperature during binder removal, and (**b**) the densification rate as a function of densification during the isothermal sintering period.

To analyze sintering behavior, the strain rate is plotted as a function of temperature in Figure 4. The binder elimination and the sintering steps were plotted separately in Figure 4a,b. The behavior during the thermal elimination of the binder is similar for all samples, which shows two prominent negative peaks. This confirms that the binder is eliminated in two steps, as reported before [44]. The first suggests the moment at which the binder is eliminated. This generates more extensive paths to eliminate the gas generated by the binder. The second peak indicates the total elimination of the larger binder, indicating that more binder is eliminated at the second stage, as shown in Figure 4a; after this, the strain rate is null, indicating that the dilatometer detects no dimensional changes.

In order to perform a better comparison among the different samples during sintering, the densification of samples has to be estimated since they differ in initial relative density. As such, the relative density during the whole sintering cycle is obtained based on linear shrinkage as follows:

The global density is determined as:

(1) $$\rho =\frac{m}{V},$$

where *ρ* is the compact’s density, *m* is mass, and is volume. Assuming that m is constant after binder elimination and during the whole densification due to sintering, the instantaneous density can be calculated as:

(2) $$\rho_{i}=\frac{m}{V_{i}},$$

where the subscript “*i*” means instantaneous density and volume, respectively. *m* is the mass measured in the sintered sample. Therefore, the instantaneous volume varies as the densification evolves during sintering, and it can be estimated from the dilatometry data. In this, the radial displacement is assumed to follow the same trend as the dilatometer measured in the axial direction. The corrective factor is the final axial-to-radial shrinkage ratio. Hence, the instantaneous volume is calculated from:

(3) Vi=(Δl+lo)(Δd+do)2(π4),

where Δ*l* and Δ*d* are determined at any instant during the thermal cycle, the relative density is defined as the compact’s weight density divided by the sample’s theoretical density, Ti64, with a theoretical density of 4.41 g/cm^3^. Thus, the relative density (D) is calculated at any instant during the whole thermal cycle by the following equation:

(4) $$D_{i}=\frac{\rho_{i}}{\rho_{t}},$$

The densification of samples indicates the volume change during sintering, and it can be estimated at any instant during the whole thermal cycle as follows:

(5) $$\frac{D_{0}-D_{i}}{D_{0}},$$

*D*_0_ and *D_i_* mean the initial and instantaneous values of the relative density. In this case, the value of *D*_0_ is taken when the sintering temperature is reached to evaluate the isothermal stage, where most of the densification is achieved. It was found that densification is similar for the three different samples at the beginning of sintering plateau and up to 10% of the densification, as shown in Figure 4b. After that, the densification rate is faster for the sample with channels of the medium size, and slowest for the sample with the smallest channels size. In all cases, the maximum value of the densification rate is reached at 10% of the densification.

The values of relative density after printing (D_g_), after the first step of binder elimination (D_b_), and after sintering (D_s_) are listed in Table 1. The value of D_g_ decreases as the channel size increases, suggesting that channel arrangement during printing allows a better distribution of the channels when they are small. This can be understood since the largest channels only have one defined channel at the center; see Figure 2. It can also be noticed that the pore volume, which is 1-D, is larger than that programmed in the printing setup. This is logical since interparticle porosity is not considered for the program. The volume fraction occupied by the channels in the sample, estimated from 3D images, confirms that samples with smaller channels are printed with high accuracy. The value of channel volume for the medium size is 21.09%, which is the closest to the intended value; as mentioned above, the printing parameters were set to fill the 80%. The sintered relative density shows the same tendency, even though the sample with channels of medium size had a larger densification during sintering.

### 3.2. Characterization Analysis

**Figure 5:**
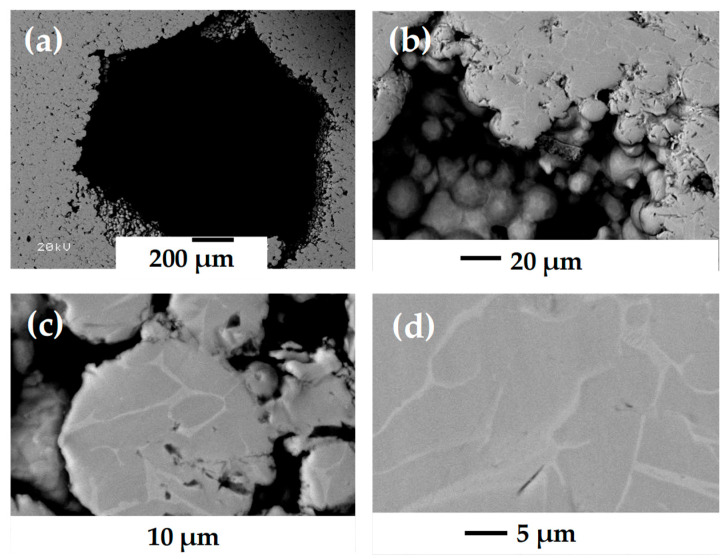
SEM images of the microstructure of sintered samples: (**a**) morphology of the channel created by printing, (**b**,**c**) necks formed between Ti64 particles, and (**d**) microstructure α and β revealed for the alloy.

Figure 5 shows the microstructure of the samples after metallographic preparation. Figure 5a shows the morphology of the channels where some deformation can be seen due to the roughing process. It can be noticed from Figure 5b that there is formation of the interparticle necks, while the spherical shape of particles remains, suggesting that sintering is in the intermediate stage. Figure 5b,c shows the formation of necks between particles, generating an interparticle porosity controlled by the sintering temperature. Finally, the microstructure corresponding to the Ti64 alloy is observed (Figure 5d), showing the β phase in the form of lamellae and its more significant proportion compared to the α phase.

**Figure 6:**
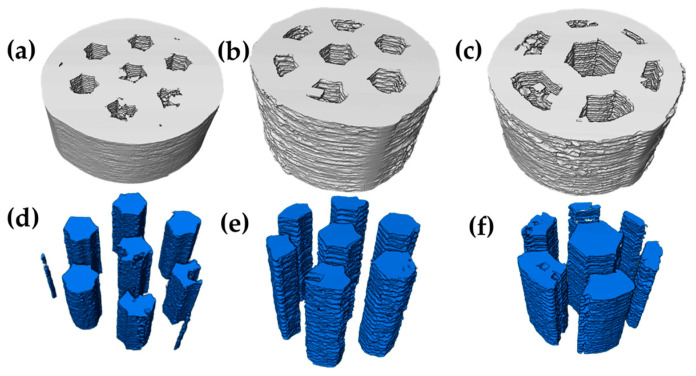
3D images of printed samples before sintering: (**a**,**d**) small channels, (**b**,**e**) medium channels, and (**c**,**f**) large channels.

Prior to the sintering process, the samples were analyzed by computed tomography; Figure 6a–c shows 3D images, where each channel size is observed, as well as its distribution throughout the sample and the morphology it has. On the other hand, Figure 6d,f shows only the channels corresponding to each size. It can be seen how the continuity of the channels is equal in the “y” direction, as well as the amount and morphology influenced by each size.

**Figure 7:**
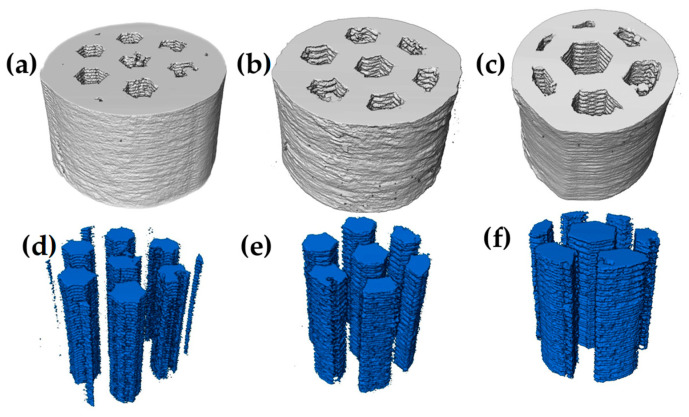
3D images of printed samples after sintering: (**a**,**d**) small channels, (**b**,**e**) medium channels, and (**c**,**f**) large channels.

After sintering, 3D images of the same samples were obtained again, showing a slight reduction in the channel size, as can be observed qualitatively in Figure 7a–c. On the contrary, Figure 7d–e, again shows the channels corresponding to each size in which no deformation of the initial shape occurs during densification, which suggests that the channel size is homogeneous in all samples.

To quantitatively evaluate the deformation of the channels during sintering, the 2D areas before and after sintering were calculated all along the height of the sample since the same channel can be directly evaluated from 3D images.

**Figure 8:**
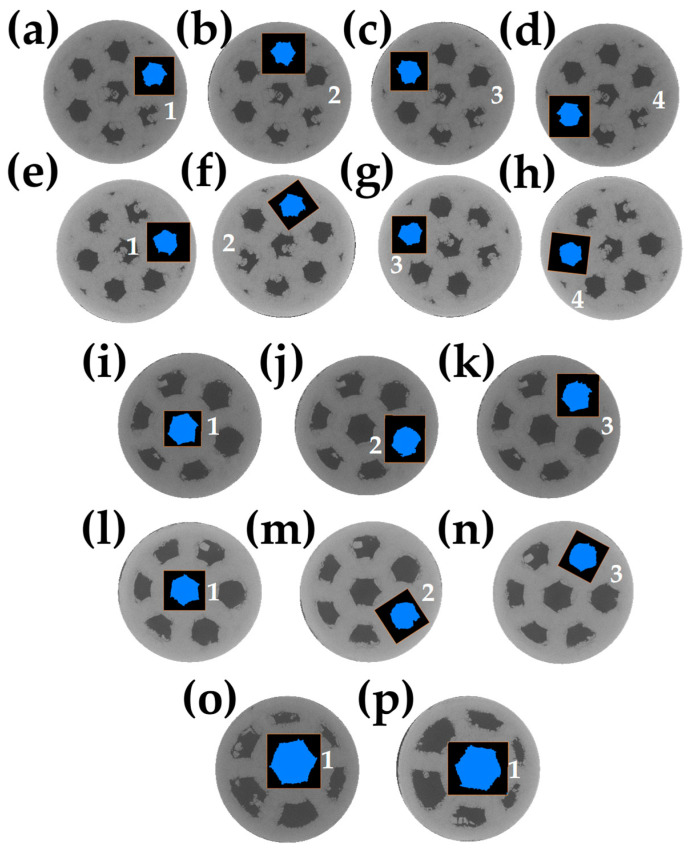
2D virtual slices before and after sintering representing the channel sectioned to obtain the area: (**a**–**d**) small channels before sintering, (**e**–**h**) small channels after sintering, (**i**–**k**) medium channels before sintering, (**l**–**n**) medium channels after sintering, (**o**) large channels before sintering, and (**p**) large channels after sintering.

Figure 8a–d shows the 2D images before sintering for the smaller size channels, Figure 8i–k for the medium-sized channels, and Figure 8o for the larger size channels with the measured channel indicated. The exact process was performed in the samples to compare the changes after sintering: Figure 8e–h for smaller sizes, Figure 8l,m for medium sizes, and Figure 8p for larger sizes. The area was obtained only in the channels that presented better morphology to obtain better results concerning the shape.

Table 2 lists the measured values of the areas before and after sintering; a reduction in the area value is found, which confirms that channels contributed to the densification of the sample. To estimate the change in the area, a similar value of the densification is calculated using the initial and final values of the areas A0 and As, respectively. This also confirms that the sample with medium channels underwent the most significant change in the area of the channel, confirming the tendency discussed above in the linear deformation and the densification. This suggests that channel deformation drives the densification of samples.

**Figure 9:**
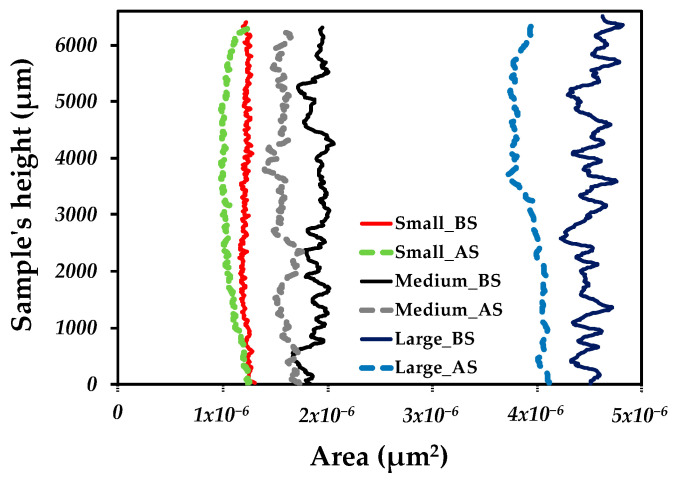
Areas obtained before and after sintering as a function of sample height.

Figure 9 plots the area values as a function of the height of the sample before and after the sintering of one channel by the sample. It can be noticed that before sintering, small channels show more or less the same value along the sample. However, medium and large channels show more irregular area values along the sample. On the other hand, after sintering, a reduction in the area values is observed for all samples. However, the sample with small channels shows a more significant area reduction in the middle of the sample, suggesting heterogeneous deformation. This behavior is different for the sample with medium and large channels, showing a more regular value of the area values along the sample instead.

### 3.3. Permeability Analysis

**Figure 10:**
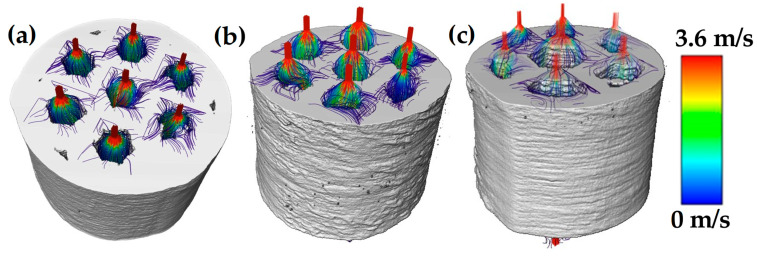
Simulated flow streamlines throughout the channels of different samples: (**a**) small channels, (**b**) medium channels, and (**c**) large channels.

3D images were used to evaluate the permeability as a function of the channel size to determine which was the most optimal compared to human bones. As such, numerical flow simulations throughout the channels were performed using the Avizo^®^ software. Figure 10 shows the simulated streamlined lines throughout the channels on the samples for the different samples. The flow is observed to pass only through the channels created during printing. Table 3 lists the permeability values for each sample, obtaining larger values as the channel size increases.

**Figure 11:**
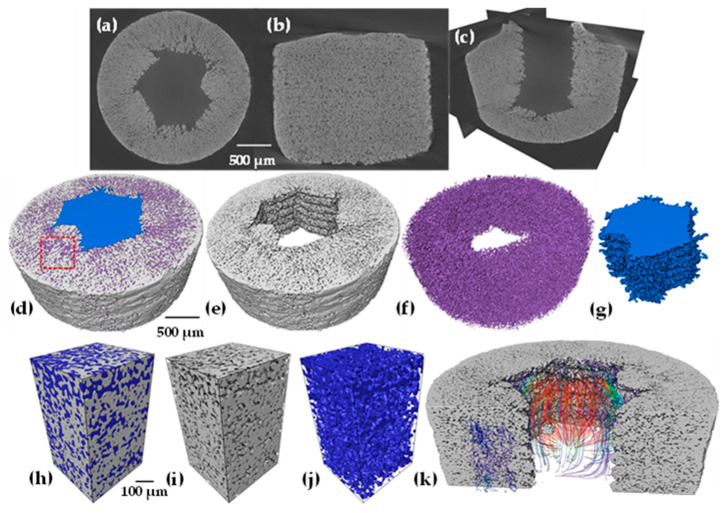
Virtual 2D slices showing the interparticle porosity (**a**–**c**), 3D rendering of the sample with small channels (**d**), particles (**e**), interparticle porosity (**f**), (**g**) the channel. 3D rendering inside and a virtual volume cropped (red square in (**d**)) showing the porosity (**h**–**j**) and simulated flow streamlines throughout the interparticle porosity and channel at high resolution (**k**).

Figure 11a–c shows a 2D image at high resolution after sintering acquired by computed tomography. It is observed in three principal planes to illustrate the defects that can be obtained during printing. It is found that the honeycomb shape is warped, which indicates that smaller shapes are too complex to be followed by the printer, as shown in Figure 11a. The printing traits as horizontal lines can also be noticed in Figure 11b. This is because the sintering temperature used did not allow considerable densification that typically reduces this kind of defects in fully dense parts [44]. On the other hand, a rendering was performed, as shown in Figure 11d, where the solid part of the sample and the porosity are observed; these elements are separated individually to obtain a better visualization. Figure 11e shows only the sintered Ti64 particles, showing the channel formed by the impression and interparticle porosity. Further, Figure 11f represents only the interparticle porosity, which is entirely interconnected. Figure 11e illustrates the channel created by printing, which highlights the roughness of the surface that is a consequence of the layer-by-layer printing process.

A virtual volume was cropped from the 3D image to illustrate the interconnectivity of the interparticle pores. This porosity is beneficial for osseointegration and helps to achieve better anchorage between the tissue–implant interface [52]. Figure 11h–j renders this 3D volume in which the porous part is separated from the solid. Finally, permeability tests were performed in the pre-fabricated channel where the flow lines have continuity; in the same way, simulations were performed in the solid part of the material and in this way to verify the interconnectivity mentioned above.

### 3.4. Mechanical Properties Analysis

**Figure 12:**
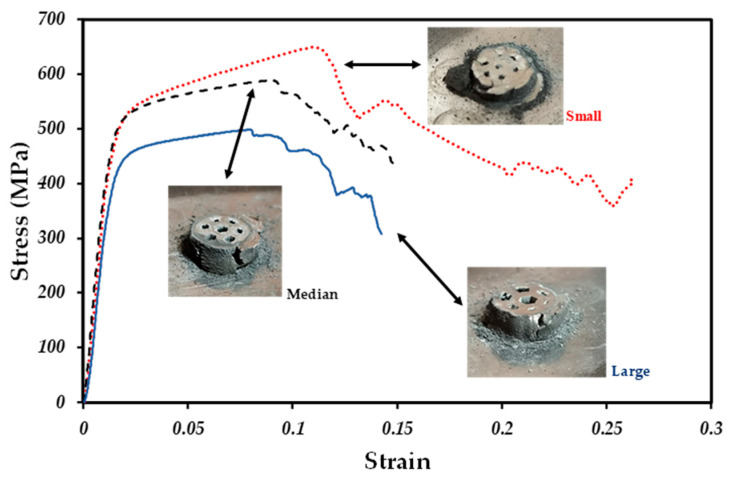
Stress–strain curves for samples with different channel sizes.

Figure 12 shows the stress–strain curves for all kinds of samples. In such curves, similar behavior is observed for the different samples during the elastic and plastic zones. However, they show a high resistance to failure; in the case of the large and medium-sized channel samples, they show the same behavior during the hardening zone until the test fails. On the other hand, the good ductility of samples can be observed in the images of fractured samples since they can be deformed entirely.

The yield stress (*σ_y_*) and the Young’s modulus (E) were calculated from the elastic section of each curve, and are listed in Table 3. The value of E is lower for larger channels, being 37.67 GPa. The yield values (394.14 MPa) follow the medium channel sizes, and finally, a higher value is obtained for the smaller channels, obtaining an E of 39.61 GPa and a *σ_y_* of 470.15 MPa.

## 4. Discussion

Samples with two different kinds of porosity were fabricated by extrusion 3D printing, followed by debinding and the prior sintering process. Samples with channels of three different sizes were designed using a honeycomb shape. The accuracy of the samples was analyzed from 3D images, finding that the geometry is not easy to reproduce as the size is reduced. This is because the angles to be followed by the printing paths are more complicated than the traditional squares studied before [27,53,54]; however, the samples were reproducible with the same accuracy. The pore volume designed for the channels was 20%, which was not reached in all samples. The smallest channels give a lower value, but the largest ones overestimate such values. This is mainly due to the distribution of the honeycomb channels on the circular surface, which induced the difference found. According to this, the sample with channels of a medium size shows the best arrangement, reaching a 21%volume. In the vertical axis of samples, the channels were perfectly straight, confirming that each layer was accurately located one over another.

The debinding schedule followed did not generate any defect or distortion of the shape of the samples. Then, sintering was carried out at a low temperature (1100 °C) with the aim to maintain interparticle porosity, which is fully interconnected according to the sintering theory and reported in the sintering of Ti6Al4V powders with a similar particle size distribution [45,47]. It is found that the channel size has an effect on the axial shrinkage of samples. The larger axial deformation measured by the dilatometry test was found for the medium-sized channels, approximately 14%. This behavior was confirmed by analyzing the change on the surface of channels by 3D images, in which the sample with medium channels also shows the largest area reduction of 16%; see Table 2. This suggests that densification during sintering is driven by the reduction in the channel volume, which is due to the sintering stresses generated during atomic diffusion and neck formation. In a general way, sintering at the particle scale is not affected by the presence of the channels as demonstrated in SEM images, in which the interparticle necks were well developed, as shown in Figure 5. The evolution of sintering was also in the intermediate stage, with interparticle porosity fully interconnected as shown in Figure 11, which confirms the fabrication of samples with two kinds of porosities that are fully interconnected among them. The graded porosity is beneficial for improving bone ingrowth, since small pores allow cell adhesion and cell proliferation, while large pores help in the vascularization to provide the nutrients required for bone ingrowth, as was demonstrated for graded porous materials of P-PEEK material [55]. This microstructure can better mimic the function of real bones, which could help to improve the osseointegration of such materials, as also reported for Ti6Al4V scaffolds fabricated by AM and implanted in rabbits [56].

Permeability was evaluated by numerical simulations from two points of view—at the sample and particle scales. This means that first numerical simulations were run on the whole samples, in which the channels were the only paths for the liquid to pass through the sample, as illustrated in Figure 10. In the second approach, numerical simulation was run on the interparticle porosity that connects with one channel, as shown in Figure 11. The values of permeability increased as the channel size did, which follows the geometrical assumptions made by Kozeny-Carman [57] and Katz and Thompson [58], where they found that as the volume fraction of pores and the pore size increase, the permeability does so too. On the other hand, the estimated values were in the range of 6.912.4 × 10^−9^ m^2^, similar to those measured for trabecular bones by Grim and Williams 0.4–11 × 10^−9^ m^2^ [59]. Those values are also in the range reported for trabecular-like porous structure materials (0.6 × 10^−9^–21 × 10^−9^ m^2^) that showed good osseointegration of such materials [60]. Thereby, permeability is directly linked to the porous characteristics as well as to the interconnection in scaffolds, which is also linked to the transport of nutrient and oxygen content. This parameter is very important since it has been demonstrated that colonization by bone cells on the scaffold surface needs different permeability values; further, the largest values improve vascularization, and lower values of permeability can enhance the cartilaginous ECM production of chondrocytes [61]. Therefore, pore connectivity in the whole sample would be beneficial to improve bone ingrowth and accelerate patient recovery.

The Young’s modulus (E) evaluated by compression tests shows similar values between 37 and 41 GPa for channels with different sizes; see Table 3. Those values are a bit higher to the ones reported for trabecular bones (1 to 22.3 GPa) that depend on the physiological and pathological conditions of bones [62]. They are also higher when compared to that of materials with higher pore volume fractions fabricated by similar processing techniques [29,63]. On the other hand, the compressive strength determined by the yield stress (*σ_y_*) ranges between 394 and 470 MPa depending on the channel size of samples, as listed in Table 3. Those values are also higher in comparison to the ones reported for the trabecular bones (5–200 MPa) by Wang et al. [64]. However, the values are in a low range compared to that of the fully dense materials actually used as bone implants and as noted, those values can be reduced by increasing the pore volume fraction in order to match the trabecular bone mechanical properties.

Table 4 lists the mechanical properties reported in different works of scaffold fabricated by AM techniques with different topologies or shapes of pores. It can be seen that most works were devoted to mimic the spongy bones because the relative density of such scaffolds is lower than 40%. Thus, the mechanical properties are low in most cases and in the spongy bones range.

**Figure 13:**
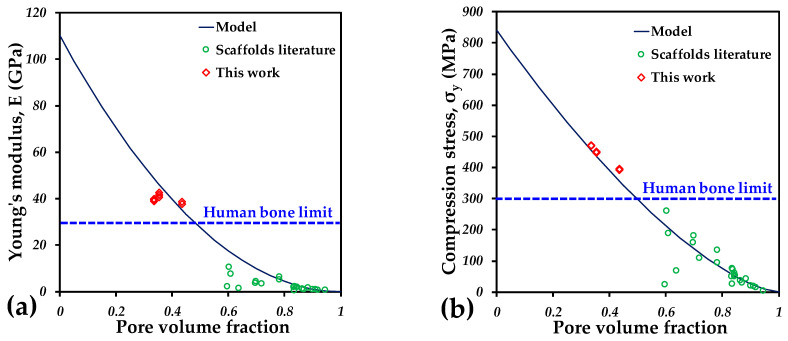
Mechanical properties of scaffolds fabricated by AM as a function of the porosity: (**a**) Young’s modulus and (**b**) yield stress.

To perform a comparison with the materials developed in this work, the Young’s modulus and the yield strength were plotted as a function of the pore volume fraction of the different samples reported in the literature and the ones fabricated in this work; see Figure 13. The behavior observed for the *E* and *σ_y_* shows a reduction as the pore volume increases, which is logical. In order to get an idea of the behavior, the model proposed by Gibson and Ashby [72] to predict the mechanical properties is also plotted in Figure 13. This model suggests that the E value is reduced in a power law as follows:

(6) $$E=\;E_{0}{\left(1-p\right)}^{2}$$

where the *E* and *E*_0_ are the Young’s modulus of the porous material and the material without pores—for this case, 110 GPa was used, which corresponds to the value of the Ti6Al4V solid alloy. Further, *p* is the pore volume fraction of samples. It was found that the Gibson and Ashby model shows good fit with the experimental values measured for the E, those of this work and those reported in the literature; Figure 13a. Similarly, the model proposed for the *σ_y_* for porous materials is similar, but with an exponential a little lower than 2:

(7) $$\sigma =\;{E\sigma }_{0}{\left(1-p\right)}^{1.5}$$

where the *σ* and *σ*_0_ are the yield strength of the porous material and the material without pores—for this case, 840 MPa was used, which corresponds to the value of the Ti6Al4V solid alloy. As shown in Figure 13b, the model can also predict the values reported very well. For both properties, the highest values reported for human bones are reported, which are lower than those obtain in this work. Nevertheless, it is also noticed that the porosity drives the mechanical properties, so, in order to reach similar values to human bones, additional porosity should be added to the materials fabricated by extrusion 3D printing. This will also provide an improvement in the permeability, which will be beneficial for the osseointegration functions and cell adhesion will always be maintained for the interparticle porosity inside the struts. This kind of porosity can be only obtained for AM techniques that involve extrusion of pellets or filaments, rather than laser techniques.

## 5. Conclusions

Cylindrical samples with honeycomb-shaped channels of different sizes were designed and fabricated by extrusion 3D printing using Ti6Al4V MIM feedstock. The shape was not precisely obtained because the angles to form the honeycomb shape are complex for the 3D printer. In spite of that, samples were accurately printed in the vertical axis, keeping the shape of channels very similar along the sample. No defects or fissures were found after the debinding and sintering processes. The main objective of this work on developing materials with two kinds of porosities was achieved, with fully interconnected channels and interparticle pores. Permeability values are in the range of trabecular bones, which is promising for the osseointegration of such materials. The mechanical properties are a little higher in comparison to that of trabecular bones; nevertheless, these properties can be tuned by increasing the pore volume fraction during the printing stage. The main conclusion is that materials with two scales of porosity can be developed by the 3D printing extrusion process.

Evaluation of cell attachment and bone ingrowth with this kind of sample is in progress to determine what size will be the most effective. This will lead to the fabrication of bone implants like hip, knees or vertebral implants, with the best size and configuration of channels and tailored porosity according to requirements.

## Figures and Tables

**Table 1:** Relative densities of samples were measured at the different stages and after sintering.

Channel Size	(D_g_) Relative Density _green_ (%)	(D_b_) Relative Density _brown_ (%)	(D_s_) Relative Density _sintered_ (%)	(D_s_−D_b_)/D_b_ (%)	Channel Volume Fraction (%)
Small	53.66	51.41	66.50	29.34	15.47
Medium	51.48	48.54	64.74	33.35	21.09
Large	49.81	45.91	56.44	22.94	24.77

**Table 2:** Areas before and after sintering and area ratio for each of the channels contained inside the samples with different-sized channels.

Channels	Area Before Sintering (E^6^)	Area After Sintering (E^6^)	(A_s_–A_0_)/A_0_
Small			
Channel 1	1.22 ± 2.96	1.06 ± 4.22	−0.13436562
Channel 2	1.22 ± 2.96	1.06 ± 7.37	−0.128123664
Channel 3	1.22 ± 2.96	1.06 ± 7.37	−0.128123664
Channel 4	1.22 ± 2.96	1.06 ± 7.37	−0.128123664
Medium			
Channel 1	1.93 ± 8.97	1.61 ± 9.62	−0.16699903
Channel 2	1.93 ± 8.96	1.61 ± 9.62	−0.16605416
Channel 3	1.93 ± 8.96	1.61 ± 9.62	−0.16605416
Large			
Channel 1	4.50 ± 1.10	1.10 ± 1.38	−0.13248393

**Table 3:** Permeability values and mechanical properties for samples with channels of different sizes.

Channel Size	Permeability (m^2^)	E (GPa)	*σ_y_* (MPa)
Small	0.69 × 10^−8^	39.61	470.15
Medium	0.86 × 10^−8^	41.74	448.60
Large	1.24 × 10^−8^	37.67	394.14

**Table 4:** Mechanical properties as a function of the relative density reported for scaffold fabricated by different AM techniques.

Topology	Relative Density (%)	E (MPa)	*σ_y_* (MPa)	Ref.
BCC	16.5–36.3	500.0–1500.0	25.9–68.6	[65]
BCCZ	5.5–40.3	750.6–2338.6	3.58–24.7	[66]
SC	9.9–13.1	1011.6–1149.1	20.2–29.3	[67]
SC	16.6–30.4	2362.9–3793.7	75.7–158.2	[68]
SC	21.9–39.2	5270.1–7565.2	94.6–188.1	[69]
Hybrid-Strut SC	15.7–30.2	1893.3–4438.5	61.8–181.6	[68]
NR-SC	11.6–16.4	1715.8–2257.5	43.2–71.6	[67]
FCC	8.1–13.7	724.2–1271.2	15.7–36.7	[70]
FCC	15.8	1930	50.2	[71]
FCCZ	9.0–15.6	1020.1–2019.1	19.7–53.0	[70]
Octahedron	16.7	1890	49.6	[71]
Octet Truss	28.2	3380	109.5	[71]
Multi-topology SC	21.8–39.7	6376.4–10,517.0	134.3–259.9	[69]

## Data Availability

The data presented in this study are available on request from the corresponding author due to the raw/processed data required to reproduce these findings cannot be shared at this time as the data also form part of an ongoing study.
